# All-optical switching in granular ferromagnets caused by magnetic circular dichroism

**DOI:** 10.1038/srep30522

**Published:** 2016-07-28

**Authors:** Matthew O. A. Ellis, Eric E. Fullerton, Roy W. Chantrell

**Affiliations:** 1 Department of Physics, University of York, York, YO10 5DD, United Kingdom; 2 Center for Memory and Recording Research, University of California San Diego, La Jolla, CA 92093-0401, USA

## Abstract

Magnetic recording using circularly polarised femto-second laser pulses is an emerging technology that would allow write speeds much faster than existing field driven methods. However, the mechanism that drives the magnetisation switching in ferromagnets is unclear. Recent theories suggest that the interaction of the light with the magnetised media induces an opto-magnetic field within the media, known as the inverse Faraday effect. Here we show that an alternative mechanism, driven by thermal excitation over the anisotropy energy barrier and a difference in the energy absorption depending on polarisation, can create a net magnetisation over a series of laser pulses in an ensemble of single domain grains. Only a small difference in the absorption is required to reach magnetisation levels observed experimentally and the model does not preclude the role of the inverse Faraday effect but removes the necessity that the opto-magnetic field is 10 s of Tesla in strength.

The ultimate switching speed of magnetic materials has long been a subject of considerable interest and debate. A faster alternative to switching using magnetic field pulses was demonstrated by Stanciu *et al*.^1^, who showed that the all-optical control of the magnetic orientation in the amorphous ferrimagnet GdFeCo could be achieved using circularly polarised femto-second laser pulses. This helicity dependent switching was first attributed to the inverse Faraday effect (IFE)^2^,^3^, where the polarised light induces a magnetic field within the media^4^. However, further investigations observed that in GdFeCo switching could take place independently of the laser helicity in what is known as thermally induced magnetic switching (TIMS)^5^,^6^. The helicity dependent switching of Stanciu *et al*. was then explained as a combination of the threshold temperature for the TIMS mechanism and magnetic circular dichroism (MCD); in that the media absorbs a different amount of energy from the light depending on the polarity and orientation of the magnetisation^7^.

Further investigations have shown that a wider range of transition metal - rare earth alloys and synthetic ferrimagnetic materials exhibit all-optical switching in some manner^8^,^9^. Intriguingly, recent experiments by Lambert *et al*.^10^ observed helicity dependent switching in ferromagnetic Co/Pt multi-layers and granular L1_0_ FePt. Until now all-optical control of the magnetisation had been confined to ferrimagnetic materials and these ferromagnetic materials exhibit high uniaxial anisotropy important for magnetic recording and other magnetic nano-technologies. The underlying physics of TIMS was investigated by Barker *et al*.^11^, who showed that the switching is caused by the excitation of two-magnon bound states. The essential requirements for TIMS are; firstly the existence of anti-ferromagnetically coupled sub-lattices and secondly the two sub-lattices must have distinct demagnetisation rates, which can be engineered through the dependence on the damping and magnetic moment. Since the ferromagnetic materials in which helicity dependent switching was observed do not satisfy these requirements it cannot be attributed to TIMS. The opto-magnetic field caused by the inverse Faraday effect remains a possible mechanism but its precise magnitude and duration are not well understood.

The role of the inverse Faraday effect in switching a continuous Co/Pt system was investigated by Cornelissen *et al*.^12^ using the microscopic three-temperature model. They conclude that the all-optical switching via the IFE is possible with an opto-magnetic field duration of 0.15 ps. However, like earlier calculations for the IFE in GdFeCo by Vahaplar *et al*.^4^, the required magnitude is approximately 20 T. This field magnitude is hard to justify unless properties such as the exchange interaction are involved but Cornelissen *et al*. claims it is within the range given by theoretical estimations. Since continuous films behave in a qualitatively different manner to granular ones and the larger uniaxial anisotropy in FePt would suggest that the all-optical switching mechanism remains unclear in these types of ferromagnets.

In this report we explore and compare two possible mechanisms for helicity dependent switching in ferromagnets; first the inverse Faraday effect and second a thermal ‘reptation’-like effect. We concentrate specifically on L1_0_ FePt for comparison with the experiments of ref. 10 which is investigated using atomistic spin dynamics^13^,^14^. Spin dynamics has proved invaluable for investigating both ultrafast laser induced magnetisation dynamics^6^,^11^ and can be specifically parameterised for L1_0_ FePt^15^,^16^. For the inverse Faraday effect, the magnitude and duration of the opto-magnetic field is varied to construct the regime in which switching is possible. Following this we present an alternative, or additional, switching mechanism; a thermal ‘reptation’-like model where switching occurs through thermal activation of the grains in analogy to Néel’s reptation model of hysteresis behaviour. In our case during a single laser pulse the grains will switch thermally and magnetic circular dichroism gives rise to a switching rate dependent on the helicity and grain polarity. Therefore, over a sequence of laser pulses a net magnetisation averaged over an ensemble of grains will reach a finite value. We utilise a 2 state Master equation model, using the switching probabilities calculated from the atomistic spin dynamics, to predict the evolution of the magnetisation as a function of increasing laser pulses. The reptation model agrees well with experiments suggesting that magnetic circular dichroism is the most plausible origin of the helicity dependent all-optical switching in granular FePt.

## Results

### Switching induced by the inverse Faraday effect

The inverse Faraday effect has been long been suggested as a mechanism for helicity dependent all-optical switching but despite various theoretical treatments^2^,^17^,^18^ its exact nature has not been fully understood. Therefore, we begin by investigating the required magnitude and duration of a opto-magnetic field, generated by the inverse Faraday effect, to cause switching of FePt grains. To determine this we employ atomistic spin dynamics; whereby localised atomic magnetic moments are evolved using the Landau-Lifshitz-Gilbert equation^13^. The model is specifically parameterised for L1_0_ FePt using the Hamiltonian derived by Mryasov *et al*.^19^; more details are given in the methods section. The heating effect of the femto-second laser is incorporated through dynamic electron and phonon temperatures that are evolved using the two-temperature model^20^,^21^. The system is equilibrated to room temperature before the laser pulse is applied. The opto-magnetic field is assumed to couple into the spin dynamics in the same manner as an applied field, that is initially zero but triggers with the laser pulse. The field is taken to have the form of a flat Gaussian with a variable central duration. To determine if switching has occurred, the system is simulated for 10 ps past the laser pulse and whether the magnetisation has passed the *m*_*z*_ = 0 plane is monitored. The large anisotropy in L1_0_ FePt is sufficient that, despite this short timescale, the magnetisation will then relax to the easy axis after the laser pulse. This is repeated 20 times to provide the probability of the grain switching from its original orientation into the reversed state.

**Figure 1:**
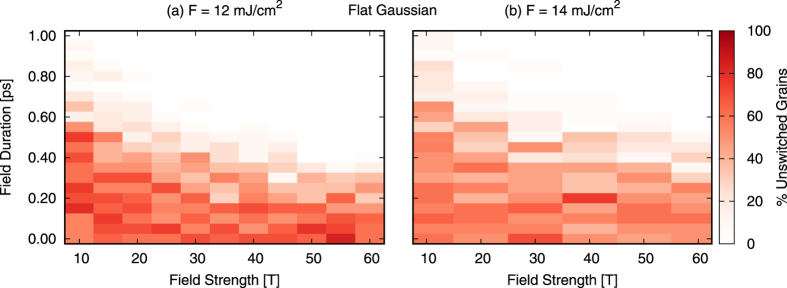
Computed switching phase space via the inverse Faraday effect. Percentage of unswitched grains using a laser fluence of (**a**) 12 mJ/cm^2^ and (**b**) 14 mJ/cm^2^ with an inverse Faraday field modelled by a flat Gaussian for varying strengths and duration. The sides of the field are Gaussian shaped with a width of *τ*_*l*_ = 100 fs.

The switching phase space, expressed as the percentage of unswitched grains, is shown in Fig. 1 for laser fluences of (a) 12 mJ/cm^2^ and (b) 14 mJ/cm^2^. There is a clear region of deterministic switching for high field strength and long duration for both fluences. However for a 10 T field magnitude a duration of approximately 0.6 ps is required while for a large 60 T field this is only reduced to approximately 0.2 ps. This represents the opto-magnetic field remaining in the material in the range of 2 to 6 times longer than the pulse width at magnitudes which are hard to produce externally. With low field magnitude and duration the field is not sufficient to cause consistent switching but there is still a possibility of switching caused by thermal fluctuations yielding a thermally demagnetised film for sufficient laser fluence. Comparing (a) and (b) we can see that the increased fluence has not improved the switching window but rather the enhanced thermal effects causes a more randomised final state. This can be seen as in the low magnitude-short duration regime the probability of remaining unswitched is reaching 50%.

This switching window is similar to that seen by Cornelissen *et al*.^12^ for Co/Pt; they find a minimum duration of 0.15 ps is required with an IFE field of 20 T. The field range is smaller than seen here due to differences in the anisotropy but the duration is comparable. Cornelissen *et al*. conclude that the IFE is a viable explanation for all-optical switching in ferromagnets but do not consider further mechanisms.

### Thermal Switching Of Grains

It is clear that the inverse Faraday effect modelled in this manner may give rise to the observed optical switching but it is also clear that thermal effects can dominate. The effect of the laser heating is to quickly demagnetise the grain within a few hundred femtoseconds but then the re-magnetisation process is on the time-scale of picoseconds^22^. This implies that within a few picoseconds after the laser pulse the magnetisation may thermally hop over the anisotropy energy barrier. This energy barrier is reduced at elevated temperatures due to magnetisation fluctuations which are naturally included in the atomistic model^23^.

**Figure 2:**
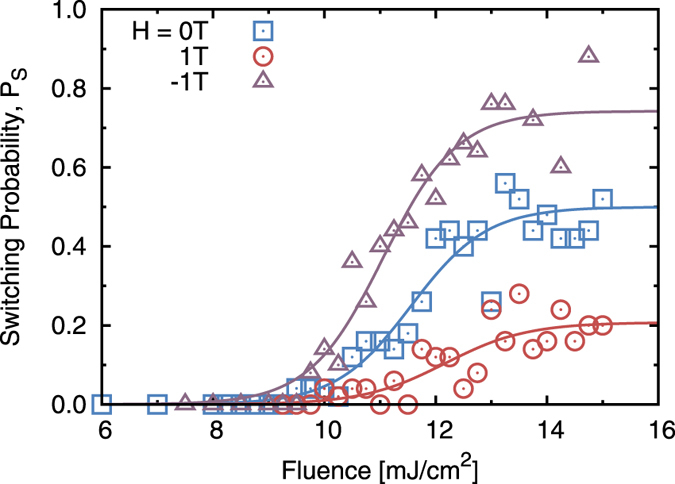
Computed probability of a single grain reversing without the inverse Faraday effect over a range of laser fluences. Even without a field the grain thermally switches when the fluence is high enough to demagnetise the grain. Above F = 16 mJ/cm^2^ the system is above the Curie temperature and will cool over a longer timescale. The switching is also calculated for a constant field of 1 T either aligned or anti-aligned with the initial magnetisation direction. The lines show a fit to the data.

To understand the role that thermal effects play we now investigate the switching probability during a single laser pulse without any opto-magnetic field as a function of the laser fluence. Figure 2 shows the switching probability averaged over 50 separate pulses in zero field and with a constant ± 1 T external field. As expected, at low fluences, below ≈ 8 mJ/cm^2^, the ratio *KV*/*k*_*B*_*T* is large and essentially the switching probability is zero. Above ≈ 8 mJ/cm^2^ there is a possibility for the grains to switch direction and the probability increases strongly with fluence until about 14 mJ/cm^2^ where the peak electron temperature is high enough to fully demagnetise the grain. At this point the grains would be evenly distributed between up and down and so the probability of switching would be 0.5. Beyond 16 mJ/cm^2^ both the electron and phonon temperatures remain above the Curie temperature on a much longer timescale governed by the cooling rate of the sample. The effect of a constant applied field is to provides a bias to the energy barrier and thus decreases or increases the switching probability depending on whether it is oriented parallel or anti-parallel to the starting magnetisation.

### Reptation model of helicity dependent switching

This dependence of the switching probability with absorbed laser fluence describes a simple mechanism for all-optical switching. If the absorbed fluence depends on the relative orientation with respect to the laser helicity then there will be a difference in the switching probability and thus a net probability of ending in a specific orientation. Since the thermal switching probability is not deterministic (*P*_*s*_ ≠ 1) the mechanism will not lead to the exact switching of that grain. Therefore we need to consider an ensemble of grains to observe the final magnetisation. In an ensemble the mechanism is as follows. The laser heats all the grains, but those grains with a magnetisation oriented in the higher absorption direction will achieve a higher temperature with consequently a relatively large switching probability according to Fig. 2. Any such grains switched will reside in the lower absorption direction, with a reduced probability of switching back. In this picture we expect a greater probability of the grain ending in the lower absorption direction.

In magnetic materials the differential absorption of circularly polarised light is known as magnetic circular dichroism and would give the required helicity dependence of this model. We note that since the difference in absorption is small the magnetisation change in a single pulse will be small. However, in the experiments performed by Lambert *et al*. the samples were subjected to a sequence of laser pulses at a repetition rate of 1kHz and consequently each grain will be excited by the laser around 1000 to 10,000 times. This is expected to lead to a continuous acquisition of magnetisation following each laser pulse; an optically-induced reptation, analogous to the magnetisation changes accumulating after a number of field pulses in Néel’s classical reptation phenomenon. In comparison to Khorsand *et al*. ’s model of helicity dependent switching in GdFeCo; TIMS provides an underlying mechanism and occurs with a single pulse while our model here is driven by thermal switching and a final magnetisation state is built over many pulses. In both cases it is the helicity which determines the final orientation of the magnetisation but the magnitudes will be different.

Within this picture we proceed to derive a simple formalism to model the effect of thermal switching with magnetic circular dichroism using a two-state Master equation. Since we are interested in granular L1_0_ FePt each grain is a single domain and due to its large anisotropy the magnetisation is fixed out of plane. Therefore we can consider the probability of each grain in the ensemble occupying either out of plane orientation. Due to the magnetic circular dichroism one of these states will absorb more energy and the other less, so we consider the probability of the grain occupying the high or low absorbing orientation; *n*_+_ and *n*_−_ respectively. For simplicity we neglect inter-granular interactions, which firstly will not affect the underlying physics involved and secondly will be of limited importance since the switching takes place at elevated temperatures. The net magnetisation, normalised to the equilibrium value, orientated in the direction of magnetisation of the low absorption state is given by *m* = *n*_−_ − *n*_+_. The time evolution of these is given by


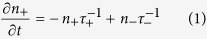



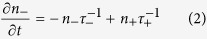


where 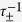 are the transition rates of the grain switching from the high (+) or low (−) absorption states to the other state. These transition rates describe the all-optical switching process that a single grain undergoes and so together these equations describe the evolution of the net magnetisation as it is subject to a series of pulses. Earlier the probability of a grain switching was calculated for a single laser pulse; we now consider a repeating laser so that the reptation effect can occur. In this case the time domain is constructed as *t* = *N*/*f*_*l*_ with N the number of the pulse and *f*_*l*_ the repeat frequency of the laser pulse. Now the transition rates as a function of the laser fluence, F, are described by


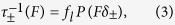


where *P*(*Fδ*_±_) is the probability of the grain switching by a single laser pulse and *δ*_±_ = 1 ± Δ/2 is a factor describing the difference in the absorbed fluence due to the magnetic circular dichroism. Δ is the MCD ratio, which is of the order of a few percent. For a detailed parameterisation of this model the switching probability is taken to be that found using the atomistic spin model in Fig. 2. A function is fitted to the data for each of the different field strengths and a linear interpolation of the fitting parameters is used; more details are provided in the methods section.

In equilibrium the states will satisfy detailed balance and the using the conservation of the total probability, *n*_+_ + *n*_−_ = 1, we find


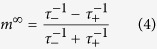



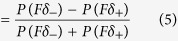


This shows the net magnetisation reached after an sufficiently long series of laser pulses. In essence this relies on the relative difference in the transition rates which can be non-zero even at very low laser fluence but may take much longer to reach.

Solving equations (1) and (2) the time dependence of the net magnetisation over a sequence of laser pulses is given by


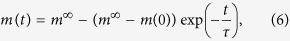


with the switching time given by


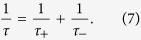


**Figure 3:**
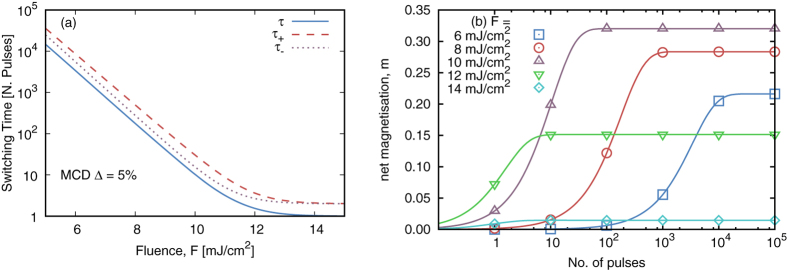
Switching times and acquisition of magnetisation over a series of pulses. (**a**) The switching times; *τ*, *τ*_+_ and *τ*_−_ as a function of fluence with a MCD of 5% expressed as number of laser pulses. (**b**) The net magnetisation with the number of laser pulses for different fluences. At low fluence the switching time is large and so many laser pulses are required to reach the final net magnetisation. At higher fluences the net magnetisation saturates within a few pulses but since thermal effects are higher the final value is lower.

The switching time, *τ*, is shown in Fig. 3(a), alongside *τ*_+_ and *τ*_−_ over a range of fluences. *τ* shows no variation with different MCD ratio while *τ*_+_ and *τ*_−_ show a widening separation for larger MCD, here only a relatively large MCD ratio of 5% is shown. The switching times drop significantly with fluence since the probability of switching shown in Fig. 2 is initially very small but follows a sharp transition to 50%. The time evolution of the net magnetisation is shown in Fig. 3(b) for an MCD of Δ = 5% calculated from equation (6). The net relaxation time depends strongly on the fluence and at low fluence the net magnetisation takes a large number of laser pulses to reach the equilibrium value. At high fluence only a few laser pulses are required to reach the equilibrium value but the magnitude is much smaller due to the stronger thermal effects.

Equation (6) can be solved numerically for any initial magnetisation state. The experiments of Lambert *et al*.^10^ were carried out on a demagnetised sample, so we consider an initial state with equal numbers of spins up and down. For this special case with, *m*(0) = 0, it is easy to show that the gradient for a small number of laser pulses is


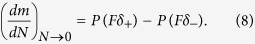


This shows that over a small number laser pulses the acquisition of magnetisation is essentially linear building up after each step. Equation (8) gives a simple approach to determine the experimental values of *P*(*Fδ*_+_) − *P*(*Fδ*_−_) which, along with the single shot switching probability, either from experiments or atomistic calculation, can be directly related to the MCD value; potentially an important check on the validity of the model.

**Figure 4:**
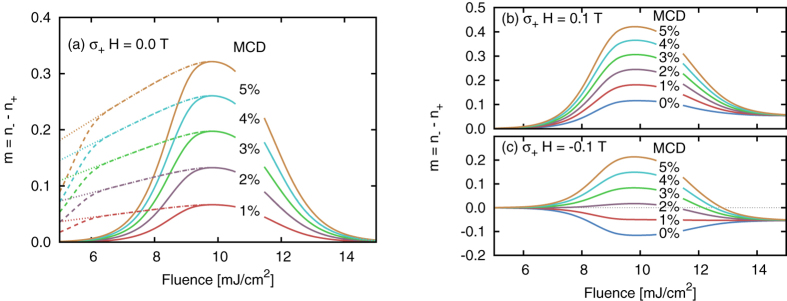
Net magnetisation after a series of laser pulses. The net magnetisation due to the MCD effect over a range of fluence and MCD percentage for (**a**) zero, (**b**) +0.1 T and (**c**) −0.1 T constant applied field strengths. The solid lines show the net magnetisation after 100 pulses while in (**a**) the dashed lines are after 1000 pulses and the dotted lines are *m*^∞^. A peak net magnetisation appears at approximately 9.5 mJ/cm^2^ and for a MCD ratio of 3% the net magnetisation is comparable to that measured by Lambert *et al*.^10^.

The net magnetisation caused by the thermal switching is shown in Fig. 4 as a function of laser fluence for a range of MCD ratios. The solid lines show the net magnetisation after 100 laser pulses and the dashed lines after 1000 while the dotted lines show *m*^∞^. Below a critical value of fluence, no acquisition of the magnetisation occurs, essentially because the laser heating is not sufficient to induce thermally activated transitions over the energy barriers. A weak dependence of the critical fluence with MCD ratio may be expected since the high absorption orientation may absorb sufficient energy to become thermally active but since the MCD ratio is small the central fluence needs to be large. Above the critical fluence the behaviour is non-monotonic, exhibiting a rapid increase resulting from increased thermal activation. At a fluence of around 9.5 mJ/cm^2^, independent of the MCD value a peak is reached, after which the magnetisation decreases due to the increasing thermal instability at elevated temperatures.

Whilst this simple model is unlikely to give quantitative results it does show that even a small MCD will give rise to a measurable net magnetisation over a series of laser pulses. The maximum net magnetisation is close to the steepest gradient of the switching probability but due to the thermal randomisation at high fluences it is not centred on the steepest part. The magnetisation in Fig. 4 is defined as in the orientation of the low absorption state and since the values are positive this implies that the system will move towards the low absorption orientation. Lambert *et al*. states that the magnetisation observed in the sample after the repeated laser excitation is approximately 20% of saturation which in our measurements is reached by a MCD of 3%. The MCD can be estimated from measurements of the polar Kerr rotation in radians which, for L1_0_ FePt with a 800 nm laser, appears to be approximately 0.87%^24^. In comparison, Khorsand *et al*. measure a MCD of 1.5% in GdFeCo and also demonstrate that it can be increased by tailoring the structure of the multilayer sample increasing up to 3%. This implies tailoring the sample would be a possible route in increasing the magnitude of the thermal switching effect.

**Figure 5:**
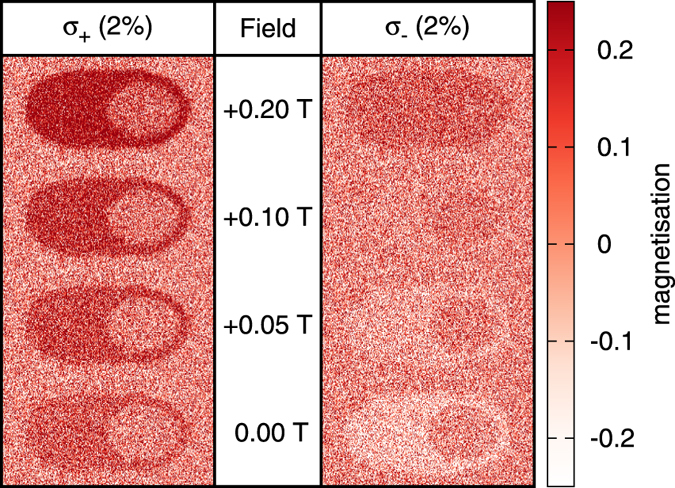
Modelled effect of a laser scan on a granular structure for various applied fields. A MCD of Δ = ±2% is used to model the laser polarisation and each grain is switched following the probabilities given in eqn (18). A Gaussian spatial profile is used for the laser with a peak fluence of 20 mJ/cm^2^ and sweeps with a constant speed.

The effect of an applied field is to bias the thermal switching probabilities, as shown in Fig. 2. Figure 4(b,c) shows the resulting net magnetisation for the case where the applied field is parallel and anti-parallel to the magnetisation of the low absorption state respectively. In (b) the field and the MCD switching cooperate so the field increases the net magnetisation. In (c) the field counteracts the effect of the MCD switching, reducing the net magnetisation. For this case the field reduces the net magnetisation to approximately 0 for for an MCD close to 2%. This agrees with the experimental results of Lambert *et al*. where a 700 Oe field could eliminate the all-optical switching. The slight difference in the field strength indicates that the true MCD value of the system is less than 2%. Figure 5 shows a more direct comparison to the experimental results where the laser is scanned across the granular sample for both helicities and various applied field strengths. A MCD of 2% is used for clarity and so the magnitude of the applied fields are slightly larger than used in the experiments but the behaviour is qualitatively the same. Exactly as Lambert *et al*. observe, the increase in the field strength improves the saturation for *σ*_+_ but for *σ*_−_ it reduces the saturation until it fully counters the optical switching at 0.1 T.

**Figure 6:**
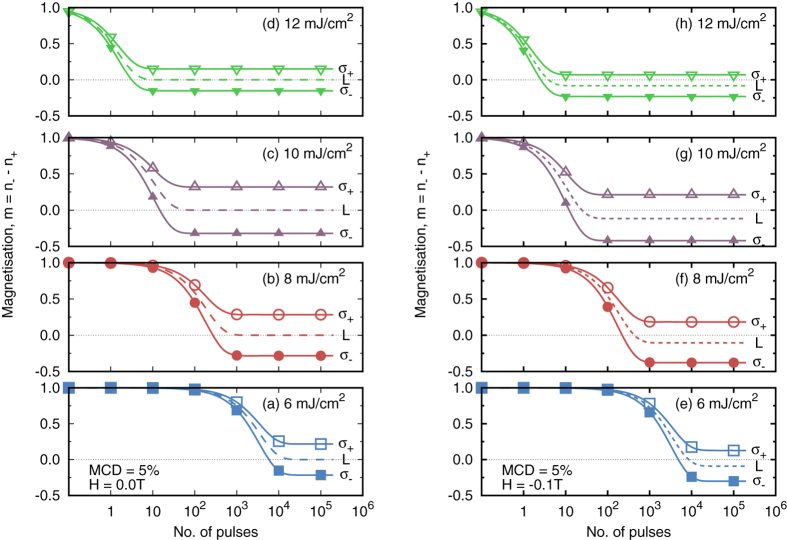
Helicity dependent evolution of the magnetisation over a series of laser pulses. The time evolution of the magnetisation starting from an initially fully saturated state with a MCD ratio of 5%. Left column (**a**–**d**), zero applied field, right column (**e**–**h**), applied field = 0.1 T applied in the negative sense relative to the initial magnetisation.

Finally we consider the effect of the helicity of the laser pulse on the evolution of the magnetisation starting from a state of full magnetic saturation. The results are given in Fig. 6, which shows the variation of the magnetisation with the number of laser pulses for both helicities. In Fig. 6 the left column shows the evolution of the magnetisation in zero applied field, and right column, the response to an applied field of 0.1 T. For comparison the case of linearly polarised light is also given for each fluence. In the case of zero applied field the magnetisation evolves to zero for linear polarisation as expected. For circularly polarised light the magnetisation initially decreases with an asymptotic approach to equilibrium values whose sign is dependent on the helicity of the polarised light. The time to equilibrium decreases with increasing fluence (a–d) while the equilibrium value first increases and then decreases. The application of a magnetic field breaks the symmetry as shown in Fig. 6(e–h). The field, in the sense applied in Fig. 6, assists magnetisation reversal, shifting the magnetisation more negative for all polarisations of the laser. These results are consistent with the more recent experimental results of Takahashi *et al*.^25^.

## Discussion

In this study, the underlying physics of optically induced switching in L1_0_ FePt media has been investigated considering two alternative mechanisms. Firstly, switching triggered by a combination of elevated temperatures and an assumed opto-magnetic field induced by the inverse Faraday effect has been investigated. By using atomistic spin dynamics parameterised from *ab initio* calculations the switching window is seen to require fields that are either of magnitude in excess of 60 T or a duration greater than 5 times that of the laser pulse. Such large fields are perhaps justifiable for a 2-sub-lattice ferrimagnet such as GdFeCo, where switching is driven by a 2-magnon bound state^11^ involving fields of the order of the exchange interaction, however, it seems less likely for a ferromagnet such as FePt.

A simpler explanation of the results presented by Lambert *et al*. is that of an optically-induced reptation. The thermal activation during the demagnetisation caused by the laser allows the grains to switch, and if the different orientations absorb different amounts of energy from the laser due to the MCD effect then there is a difference in the transition rates. In a simple 2 state Master equation approach, using single-shot transition probabilities determined by the atomistic model, these different transition rates are shown to give rise to an net magnetisation over repeated cycling of the laser. An MCD of 3% is sufficient to induce a magnetisation similar to that seen experimentally. The effect of an applied field is to bias the transition probabilities and which then inhibits the reptation effect when it is parallel to the magnetisation of the high absorption state or aids it when in the opposite orientation. This model of the helicity dependent all-optical switching in FePt seems physically justifiable and requires only the assumption of an MCD value of Δ ≈ 2%. However, it does not exclude the IFE from playing some role in the switching but importantly it removes the necessity to invoke the unreasonably large opto-magnetic fields required for the IFE to be the dominant mechanism. Finally, we have shown that optically-induced switching over a series of laser pulses, the optical reptation effect, is a new mechanism distinct from TIMS in granular ferromagnets. Although the effect is relatively weak, as found experimentally by Lambert *et al*.^10^, it is likely that optimisation of material parameters using the ideas presented here could lead to a new approach to energy assisted magnetisation reversal.

## Methods

### Atomistic spin dynamics

Atomistic spin dynamics models the magnetic material as Heisenberg (classical) spin moments that are localised to the atomic sites. The ensemble of spins are modelled by the time integration of the Landau-Lifshitz-Gilbert equation:


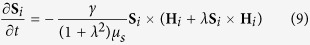


where the S_*i*_ = *μ*_*i*_/*μ*_s_ are three-dimensional reduced magnetic moments of unit length and the effective field is 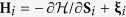. *λ* is the atomistic damping (coupling) and ***ξ*** is a stochastic thermal noise term which is used to keep the ensemble at a finite temperature. The thermal noise is a Gaussian white noise process with the following mean and variance:


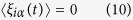



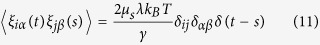


To integrate the coupled equations of motion the semi-implicit method is used^26^,^27^ which is integrated using a time step of Δ*t* = 1 × 10^−16^ s. To accelerate the dynamics the model is implemented on graphics processing units (GPUs).

FePt is modelled in the ordered L1_0_ phase using an effective spin Hamiltonian which was constructed by Mryasov *et al*.^19^ on the basis of first-principles calculations of non-collinear configurations using constrained local spin density functional theory. It was found that magnetic interaction parameters are strongly affected by the fact that the magnetic moment of the Pt sites is entirely due to the exchange fields provided by the Fe sites. It was shown that this important feature of the electronic interactions can be taken into account within a model of localised Fe magnetic moments with modified effective magnetic interactions. The full Hamiltonian, described in detail in ref. 19, is


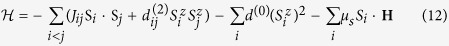


The first sum represents the Heisenberg exchange energy of magnetic moments and contains both an isotropic exchange and two-ion anisotropy component that are not restricted to nearest-neighbour interactions. Consequently the exchange interactions *J*_*ij*_ (and also 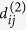) have to be taken into account up to a distance of 5 unit cells until they are finally small enough to be neglected. The two-ion anisotropy parameters 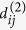 are the dominant contribution to the uniaxial anisotropy energy in relation to the single-ion term *d*^(0)^ which is represented in the second sum.

### Incorporating the femto-second laser

The effect of the laser is incorporated using the two temperature model^20^ where the electrons and phonons exist as distinct heat baths in quasi-equilibrium. The laser couples directly to the electron heat bath which then transfers energy to the phonon and spin system. In this case the spin thermal noise above is coupled to the electron temperature and the spin ensemble itself represents the spin heat bath and no separate temperature is assigned to it. The temporal evolution of the electron, *T*_*e*_, and lattice temperatures, *T*_*l*_, are governed by the following equations^28^:


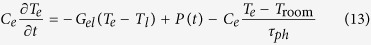



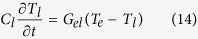



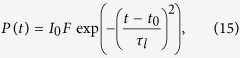


where *C*_*e*_ and *C*_*l*_ are the electron and phonon heat capacities respectively and *G*_*el*_ the electron-phonon coupling constant. *P*(*t*) describes the laser heating power which has a Gaussian shape centred at *t*_0_ with the pulse width *τ*_*l*_ = 100 fs. The fluence, *F*, is coupled through a material specific constant *I*_0_. Parameters for granular FePt are given by Mendil *et al*.^28^ which are extracted from comparison to experimental results.

Theoretical treatments of the inverse Faraday effect predict that the interaction of the polarised light with a magnetised media will create a magnetisation or magnetic field within the media^3^,^17^. Following the description given by Kimel *et al*.^29^ the resulting field depends on the electric field of the laser, **E**, as


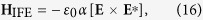


where *ε*_0_ is the vacuum dielectric constant and *α* is the coefficient of the magnetisation linear term in the expansion of the dielectric tensor *ε*_*ij*_. The direction of the field is either parallel or anti-parallel to the propagation direction, depending on the helicity, and so we consider a perpendicular set up where this is along the film normal, i.e. 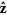. Whilst there is various theoretical predictions for the strength of this field we leave this as an open parameter. Thus, the opto-magnetic field utilised in the spin dynamics simulations is


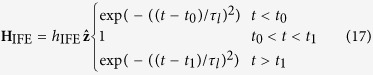


where *h*_IFE_ and *t*_*d*_ = *t*_1_ − *t*_0_ are the magnitude and duration of the IFE field respectively. *t*_0_ and *τ*_*l*_ are the centre and pulse width of the laser in the same manner as used to model the heating effects of the laser.

### Probability of switching extracted from atomistic spin model

To parameterise the two state Master equation the probability of a single grain switching is calculated using atomistic spin dynamics. The results are shown in Fig. 2; and to provide a functional form the following equation is fitted to each of the data sets


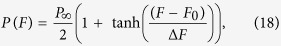


with *P*_∞_, *F*_0_ and Δ*F* as fitting parameters. With no external field the effect of a sufficiently high fluence will be to demagnetise the grain completely and as *F* → ∞ the grain will have an equal chance of entering either orientation meaning *P*_∞_ = 1/2. With a constant external field the switching will be biased but thermal effects mean that not all of the grains will align with the field as the system cools implying that *P*_∞_ for *H* ≠ 0 will only tend to 1 for high fields. Since fitting was done for zero field and ±1 T to model the effects of a smaller field a linear interpolation of the constants in equation (18) is used.

## Additional Information

**How to cite this article**: Ellis, M. O. A. *et al*. All-optical switching in granular ferromagnets caused by magnetic circular dichroism. *Sci. Rep.* **6**, 30522; doi: 10.1038/srep30522 (2016).
